# The effectiveness of physiotherapy-led non-surgical and perioperative interventions for glenohumeral osteoarthritis: A systematic review

**DOI:** 10.1177/17585732261450961

**Published:** 2026-05-22

**Authors:** Daha Garba Muhammad, Nadine E Foster, Mickylle Pelaez, Karina O’Leary, Ilana N Ackerman, Jonathan G Quicke

**Affiliations:** 1 Surgical Treatment and Rehabilitation Service (STARS) Education and Research Alliance, 1974 The University of Queensland and Metro North Health, Brisbane, Australia; 2 Centre for Innovation in Pain and Health Research (CIPHeR), School of Health and Rehabilitation Sciences, The University of Queensland, Queensland, Australia; 3 Independent Researcher, Vancouver, British Columbia, Canada; 4 School of Public Health and Preventive Medicine, Monash University, Melbourne, Australia

**Keywords:** effectiveness, exercise, glenohumeral osteoarthritis, physiotherapy, rehabilitation, systematic review, shoulder

## Abstract

**Background:**

Physiotherapists are commonly involved in the management of adults with glenohumeral osteoarthritis (GHOA), to improve symptoms or restore function after surgery. This systematic review investigated the effectiveness of physiotherapist-led interventions in adults with GHOA including rotator cuff arthropathy (RCA).

**Method:**

We conducted a comprehensive systematic review (PROSPERO Registration**:** CRD42022318534) searching PubMed, Cochrane, CINAHL, EMBASE and PEDro databases from inception up until June 2025. Articles were included if they were randomised controlled trials (RCTs) involving adults aged ≥45 years with GHOA including RCA across non-surgical and perioperative settings where interventions were led by physiotherapists and articles reported pain or functional outcomes, or adverse events. Results were narratively synthesised.

**Results:**

Of 582 articles identified, four RCTs were eligible and included in the review. Each focussed on postoperative physiotherapist-led interventions and none were placebo-controlled. Low-certainty evidence showed no between-group differences in patient outcomes at any time point between postoperative physiotherapist-led interventions delivered early (within the first 6 weeks) versus delayed interventions delivered after 6 weeks. Evidence from the remaining findings of this review is of very low certainty.

**Conclusion:**

The evidence base about the effectiveness of physiotherapist-led interventions for GHOA and RCA is extremely limited and of poor quality. No published RCTs have evaluated physiotherapist-led interventions for patients with GHOA undergoing non-surgical care. High-quality, adequately powered RCTs are required to determine effectiveness of physiotherapist-led interventions for GHOA and RCA and inform evidence-based care.

## Introduction

Osteoarthritis (OA) is the most common form of arthritis and a leading cause of musculoskeletal disability.^1^ The glenohumeral (GH) joint is the third most common large joint affected by OA, with primary GHOA affecting approximately 16% to 20% of adults aged 65 years and above.^2,3^ An untreated massive rotator cuff tear can also progress to secondary GHOA, which is referred to as rotator cuff arthropathy (RCA).^4^ Both primary GHOA and RCA are associated with activity-dependent pain that can severely impact an individual's physical function, mental health, sleep and overall quality of life.^2,5,6^ Primary GHOA and RCA are the leading reasons for shoulder arthroplasty, an increasingly common procedure internationally.^7–15^ While shoulder arthroplasty is considered the definitive treatment for GHOA, it is not without complications such as risk of failure, infections and revision surgery,^16^ and does not always lead to satisfactory results, particularly in younger and active adults.^17^ Additionally, shoulder arthroplasty is a costly procedure, for example, shoulder arthroplasty accounted for an annual societal cost of up to $1.8 billion in the USA in 2018^18^ and is projected to cost AUD$1.46 billion in Australia by 2035.^15^

General OA clinical guidelines recommend education, exercise, and self-management for primary GHOA,^19,20^ and these interventions are commonly led by physiotherapists. Where surgery is unavoidable, physiotherapists are also involved in the perioperative stage to help in restoring function.^21^ However, the evidence base underpinning these guidelines has focussed mostly on the effectiveness of these interventions for knee, hip or hand OA,^22–25^ and very few studies have focussed on GHOA.^26^ Weak evidence and expert opinion are conflicting about the effectiveness of physiotherapist-led interventions for adults with symptomatic GHOA.^27–31^ Only two GHOA-specific clinical practice guidelines, which are likely now outdated, concluded there was insufficient evidence to support the recommendation of physiotherapist-led interventions for GHOA.^32,33^ Existing systematic reviews have focussed on either conservative care broadly,^34,35^ or postoperative rehabilitation alone^36,37^; or included a mix of studies including non-physiotherapist-led interventions.^35–37^ Furthermore, nearly all of these reviews conducted their database searches over 5 years ago^35–37^ and due to the limited evidence base new studies are likely to influence the conclusions made. A contemporary, high-quality systematic review of randomised controlled trials (RCTs) is required to synthesise the best evidence for the effectiveness of physiotherapist-led interventions and highlight gaps requiring future research. Therefore, in line with the 2024 Arthritis Foundation (AF) and American Orthopaedic Society for Sports Medicine (AOSSM) GHOA Think Tank recommendation,^4^ this systematic review aimed to determine the effectiveness of physiotherapist-led interventions in reducing pain and improving function in patients with GHOA, including RCA, from RCTs published to date.

## Materials and methods

### Protocol registration

This systematic review was prospectively registered at the International Prospective Register of Systematic Reviews (PROSPERO) (CRD42022318534) and adheres to the Preferred Reporting Items for Systematic Reviews and Meta-Analysis (PRISMA)^38^ and Synthesis without meta-analysis (SWiM)^39^ guidelines for reporting. Supplementary files 1 and 2 provide the PRISMA and SWiM checklists, respectively.

### Search strategy and study selection

A comprehensive search was conducted using a combination of keywords and MESH terms for shoulder and OA, physiotherapy, pain and function and the Cochrane strategy filter for RCTs^40^ as shown in Supplementary file 3. The search was adapted and run across the following databases from their dates of inception until June 2025: PUBMED, EMBASE, Cochrane Central Register of Controlled Trials (CENTRAL), CINAHL, and PEDro. These databases were selected as they are the most comprehensive databases indexing RCTs of physiotherapist-led interventions.^41^

Articles were eligible for inclusion if: they reported the results of RCTs; they included adults (aged 45 years and over) with GHOA, confirmed either by radiographic examination or clinical evaluation, irrespective of whether the participants had primary GHOA or RCA; they were of any type of intervention as long as it was led by a physiotherapist compared with any other comparison or control; they focussed on either conservative, preoperative or postoperative care; and reported an outcome measure of pain and/or function and/or adverse events (AEs). Table 1 details these criteria. Where articles included mixed participant samples, they were included if at least 80% of the total sample had primary GHOA or RCA or the combination of GHOA and RCA participants constituted 80% or more of the total sample. No restriction on language was applied to the database searches.

**Table 1.** Summary of inclusion and exclusion criteria.

Inclusion criteria	Exclusion criteria
**Study designs** Randomised controlled trials (RCTs)	Any study design other than RCTs such as quasi-randomised, cohort studies etc.
**Participants** Adults aged 45 years and over with a diagnosis of shoulder osteoarthritis (glenohumeral joint) confirmed by imaging findings or from a clinical diagnosis.	Studies on participants with other types of arthritis such as rheumatoid arthritis. Studies with mixed samples were excluded when less than 80% of the total sample have primary GHOA or RCA or the combination of GHOA and RCA constitutes less than the 80%.
**Intervention** Any physiotherapist-led intervention in all settings.	
**Comparator** Other forms of physiotherapist-led interventions Other forms of treatment (eg. medication, usual care, surgery, injection etc) Control (eg. no treatment, waiting list, attention control, placebo therapy)	If intervention groups receive identical physiotherapy interventions Interventions routinely used by physiotherapists but not led by a physiotherapist such exercise interventions for GHOA delivered by occupational therapists etc.
**Outcome** Contains at least one measure of pain/function/ adverse events	
**Publication type** Peer reviewed journal articles	Conference paper Thesis etc.

After removal of duplicates (using EndNote), two reviewers (DGM and JQ) independently screened titles and abstracts in Covidence to identify potentially eligible articles and then full texts to screen potential articles against all eligibility criteria. Disagreements were resolved through discussion between the two reviewers. Forward and backward citation searching of included RCTs was also conducted to identify other potentially relevant RCTs.

### Data extraction

Data extraction was undertaken by one reviewer (DGM) and independently double-checked by another reviewer (JQ). The data extraction sheet was designed in Excel to capture trial identification, participant data, intervention and comparison/control group data, and outcomes data. We adapted self-reported pain and function as the primary outcomes from OMERACT-OARSI recommendations for lower limb OA.^42^

In the absence of an established Cochrane hierarchy of outcome measures for GHOA we prioritised extracting items from (in hierarchical order): The Shoulder Pain and Disability Index (SPADI) because it has been shown to be valid and responsive to change in studies of physiotherapy and shoulder arthroplasty,^43^ then, the American Shoulder and Elbow Surgeon's Score (ASES), a widely used outcome in postoperative rehabilitation, and then the Disabilities of the Arm, Shoulder and Hand (DASH),^36^ outcome measure which is validated for use in physiotherapy.^43^ Additionally, the visual analogue scale (VAS) is the most widely used pain assessment scale and therefore we extracted VAS pain scores were reported.^44^ We planned to extract (where possible) means and standard deviations of the outcome measures and sample sizes from the RCTs to allow for effect size estimation, otherwise all relevant metric available were extracted (e.g. means or *p*-values, where these were the only data available). To align with other high-quality systematic reviews of non-surgical OA management,^45–47^ we categorised the outcome follow-up period into three time-points: short-term (outcomes reported at or close to 12 weeks), medium-term (outcomes reported at or close to 6 months), and long-term (outcomes reported at or close to 12 months). We also extracted adverse event data from each RCT.

### Risk of bias and certainty of evidence evaluation

Two independent reviewers (DGM and any of NEF, MP, KOL, or IA) assessed the risk of bias assessment using the Cochrane Risk of Bias (RoB 2.0) tool^48^ with disagreement resolved through discussion between the reviewers. The certainty of evidence was evaluated in GRADEpro GDT using Grading of Recommendations Assessment, Development and Evaluation (GRADE).^49^ This evaluation was done by DGM and independently doubled checked by JQ.

### Data synthesis

Initially, a meta-analysis was planned but due to methodological and clinical heterogeneity of the included RCTs, such as intervention type, comparator and outcome reporting, narrative synthesis was deemed more appropriate.^39^

Narrative synthesis was conducted by grouping the interventions together based on the focus of the intervention such as early rehabilitation which entailed initiation of postoperative rehabilitation immediately after a shoulder arthroplasty procedure or delayed commencement of rehabilitation.^50^ Where possible, standardised effect estimates in the form of mean differences (MD), 95% confidence intervals (CIs) and associated *p*-values were calculated using MedCalc Software Ltd. Where it was not possible to calculate this standardised effect estimate, ‘no information’ (NI) was noted. Using the ‘vote counting’ approach, for each outcome, we compiled a table and narrative summary of RCTs that reported each direction of effect and the number of RCTs that found either benefit (‘favouring the intervention group’) or no benefit (‘no difference’ or ‘favouring the comparator’), designated as ‘+ve’, ‘-ve’ and ‘0’, respectively. This approach seeks to identify any evidence of the effect of an intervention over a comparator tested using a two-sided binomial probability test.^51^ The associated CI was calculated using Clopper–Pearson (exact).^52^ The included RCTs are presented and ordered in a table based on their risk of bias, as advocated by Cochrane.^51^

Severity and frequency of adverse events were also summarised from included RCTs.^53,54^ We categorised adverse events into mild, moderate or severe, where mild refers to an adverse event that is bothersome but not requiring a change in treatment, moderate adverse events require a change in treatment, additional therapy or hospitalisation, and severe adverse events are those that are disabling or life-threatening.^53^

## Results

**Figure 1.**
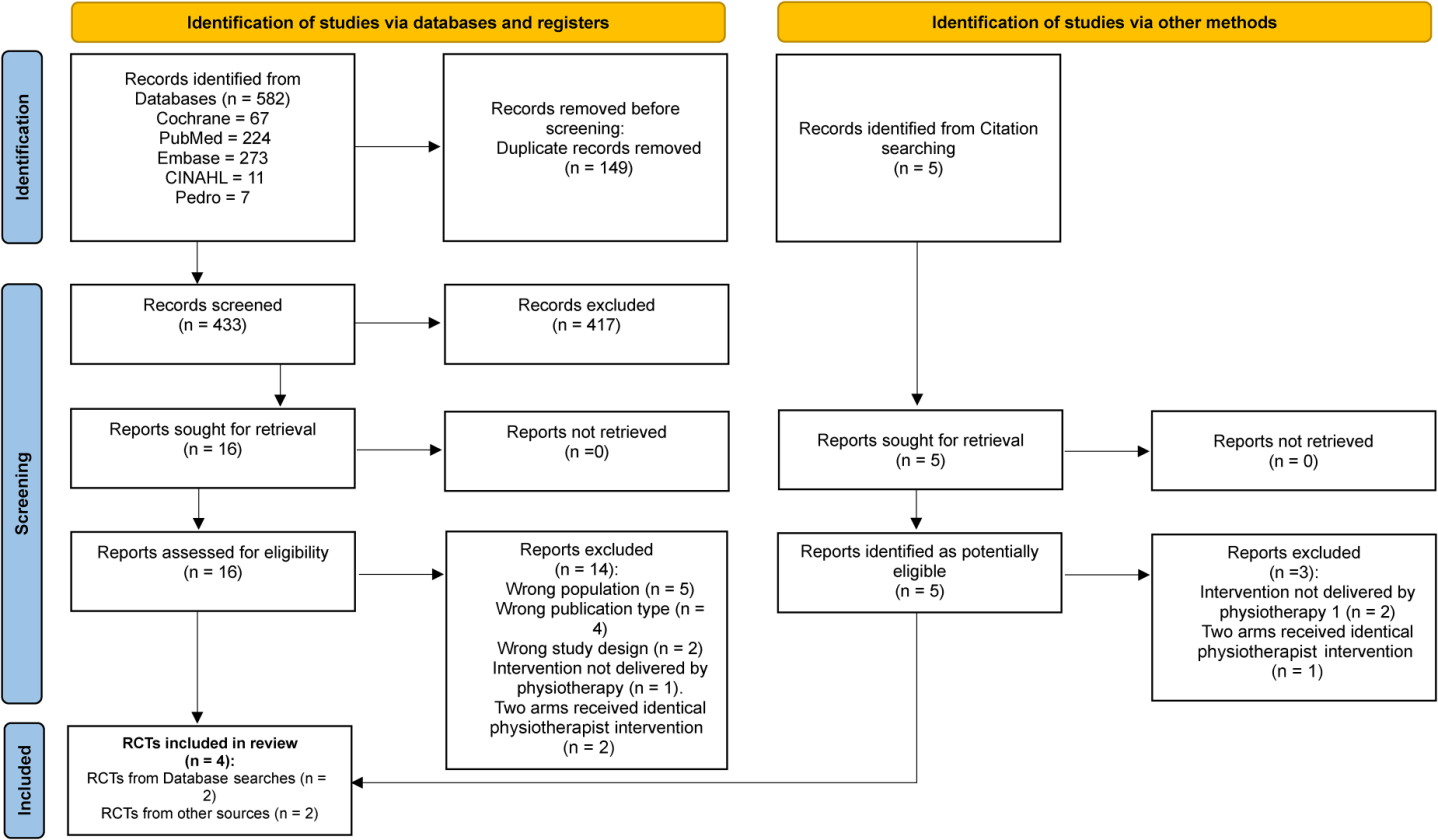
PRISMA study search flow chart.

Figure 1 shows the PRISMA study search flow chart. A total of 582 articles were retrieved from the database searches, and the full texts of 16 articles were screened against the eligibility criteria, with two meeting the inclusion criteria.^55,56^ Five potential RCTs were further identified through citation searching^57–61^; two of which met the inclusion criteria.^57,58^ Overall, four RCTs met the inclusion criteria and were included in the narrative synthesis. The excluded “near miss” and ongoing RCTs are presented in Supplementary file 4.

### RCT characteristics

Table 2 describes the four included RCTs. All were conducted in the USA and all focussed on postoperative care after total shoulder joint arthroplasty, with no RCTs focusing on physiotherapist-led interventions for patients with GHOA not having surgery. The four RCTs randomised a total of 294 participants (range 36–103), with a mean age ranging from 65.6 to 70.5 years.

**Table 2.** Description of included studies.

Trial identification (RCT first author, year of publication, country, language, care setting stage, participants number (*N*))	Participant details (surgery, diagnosis method, GHOA samples (*n*), mean age)	Intervention group	Control group	Outcome domains	Outcome measures (follow-up time points: short (S), medium (M) and long (L))
Hagen et al., 2020^58^; USA; English; postoperative care; *N* = 103	RTSA; NI, *n* = 74 (86%) RCA; mixed sample; 68.9 years	Immediate rehabilitation	Delayed rehabilitation	Pain, function and AEs	ASES pain and ASES composite at S, M and L
Khalil et al., 2023^56^; USA; English; postoperative care; *N* = 66	ATSA; NI, *n* = 66 (100%) GHOA; GHOA only; 65.9 years	Early subscapularis rehabilitation	Traditional rehabilitation	Pain, function and AEs	ASES composite at S, M and L
Baumgarten et al., 2018^57^; USA; English; postoperative care; *N* = 36	ATSA; Radiographic, *n* = 36 (100%); GHOA only; 70.5 years	Neutral rotation sling position	Internal rotation sling position	Pain and function	VAS and DASH at S, M and L
Chalmers et al; 2023^55^; USA; English; postoperative care, *N* = 89	RTSA**;** NI, *n* = 27 (30%) GHOA + 68 (65%) RCA; mixed sample; 70.3 years	Active supervised multimodal physiotherapy	Structured home exercise program	Pain, function and AEs	VAS; ASES composite at S and L; Adverse events

RCA* *=* *Rotator cuff arthropathy, ATSA* *=* *Anatomical total shoulder arthroplasty, RTSA* *=* *Reverse total shoulder arthroplasty, NI = no information, VAS* *=* V*isual Analogue Scale, ASES* *=* *American Shoulder and Elbow Surgeons Score, DASH* *=* *Disabilities of Arm, Shoulder and Hand, AEs* *=* *adverse events, S = short-term time point (12 weeks), M = medium-term time point (6 months), L = long-term time point (12 months).

Two RCTs^56,57^ recruited participants with a diagnosis of GHOA only who underwent anatomic total shoulder arthroplasty (ATSA). The other two RCTs recruited participants with mixed diagnoses of GHOA, RCA and other conditions who all underwent reverse total shoulder arthroplasty (RTSA).^55,58^ Only one RCT reported the GHOA diagnosis method, which comprised radiographs.^57^

The physiotherapist-led interventions varied across the four RCTs. Three compared one physiotherapist-led intervention to another,^56–58^ and one RCT compared a physiotherapist-led intervention to an unsupervised home exercise program (HEP) after RTSA.^55^ Khalil et al.^56^ compared subscapularis-specific rehabilitation versus traditional rehabilitation after ATSA, Hagen et al.^58^ compared early versus delayed rehabilitation after RTSA.^56,58^ One RCT^57^ compared the outcome of using a sling in a neutral position to a sling in an internally rotated position after ATSA.

Pain outcomes were reported using a VAS in two RCTs^55,57^ and the ASES pain subscale Hagen et al.^58^ in one study.^58^ One study did not report a pain outcome.^56^ Physical function was reported in three RCTs using the ASES composite score and in one RCT using the DASH score.^57^ Adverse events were reported in three RCTs^56–58^; including mild (such as Nerot-Sirveaux class), moderate (such as instability) and severe (such as embolism). However, none of the RCTs differentiated between adverse events resulting from physiotherapist-led interventions or from the surgery nor did they classify the severity of the adverse events.

### Effects of interventions

#### Early versus delayed rehabilitation after total shoulder arthroplasty

**Figure 2.**
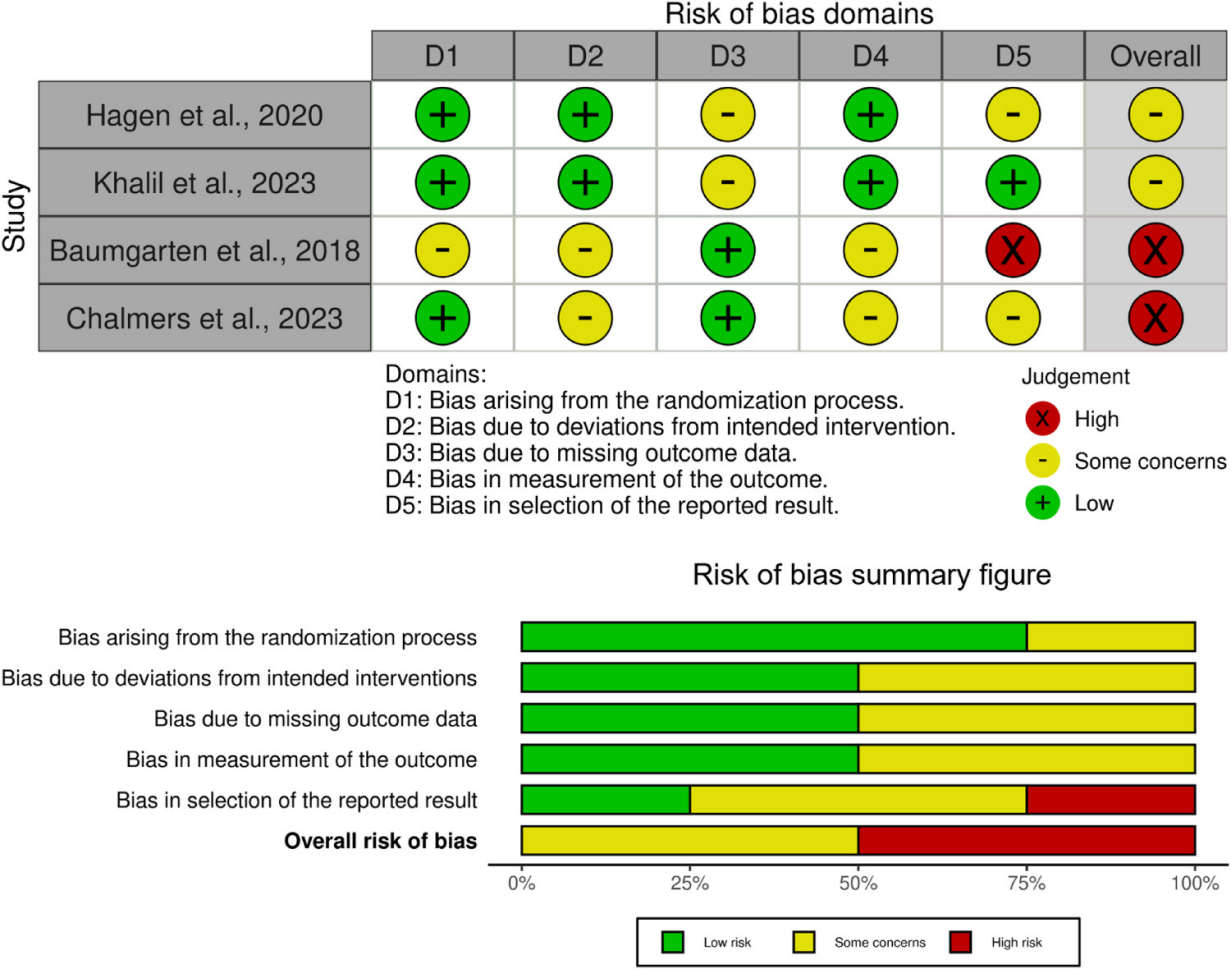
ROB assessment.

Two RCTs with a total of 169 participants provided data for this comparison.^56,58^ Khalil et al.^56^ recruited primary GHOA participants and defined early rehabilitation based on the presence or absence of additional early subscapularis exercises in the first 4 to 6 weeks after ATSA. One group the subscapularis rehabilitation (early) group received, in addition to traditional therapy, early subscapularis exercises, and the traditional (delayed) rehabilitation (TR) received traditional therapy only. Hagen et al.^58^ recruited a mixed sample including participants with primary GHOA and RCA and defined immediate (early) rehabilitation as involving immediate physical therapy for passive and active ROM and weaning of sling use as tolerated but no resistance training for 6 weeks. Delayed rehabilitation was defined as sling immobilisation with no passive or active motion of the shoulder for 6 weeks. Both RCTs showed no direction of effects (100% (binomial exact two-sided 95% CI: 16%–100%), *p* = 0.50), therefore this low-certainty evidence showed no clear evidence of direction of effects at short, medium or long-term time points (Table 3). Although in one RCT^58^ the results favoured the intervention group in terms of pain reduction in the medium term (100% (binomial exact two-sided 95% CI: 25%–100%), *p* = 1.0), the evidence for a clear direction of effects cannot be ascertained. Both RCTs were judged to have ‘some concern’ in the risk of bias assessment (Figure 2). The available effect estimates are presented in Table 3.

**Table 3.** Summary of intervention effect and certainty of evidence.

Outcome (no. of studies) Effects estimate from individual studies	Vote counting (direction of effects)	Certainty of evidence^†^
**Early versus Delayed rehabilitation** ^¥^		
***Pain (1) (number of participants = 103)*** CRITICAL **Hagen et al., 2020**^58^ (ASES pain, 0–50 (mean change)),* *higher = better* **Short-term:** NI, **Medium-term:** MD* *=* *9.6, **Long-term:** NI	**Short term:** 0 (1), **Medium term:** 0 (1), **Long term:** 0 (1)	⊕⊕◯◯ Low^a,b^
***Function (2) (number of participants = 169)*** CRITICAL **Hagen et al., 2020**^58^ (ASES composite, 0–100 (mean change))* *higher *=* better* **Short-term:** NI, **Medium-term:** MD* *=* *10.2, **Long-term:** NI **Khalil et al., 2023**^56^ (ASES composite, 0–100)* *higher *=* better* **Short term:** −5.9 [−17.3, 5.5], *p *=* *0.30, **Medium-term:** 1.2 [−11.7, 14.1], *p *=* *0.9, **Long-term:** −13.6 [30.3, 3.05], *p *=* *0.1	**Short term:** 0 (2), **Medium term:** 0 (2), **Long term:** 0 (2)	⊕⊕◯◯ Low^a,b^
***Adverse events (2)(number of participants**** *=* ****169)*** IMPORTANT **Hagen et al., 2020**^58^ **Delayed rehabilitation: Frequency**: Rare/ 4 AEs, **Severity**: *Severe: 2* (thromboembolism, and of lymphedema), *Moderate*: 2 (prosthetic shoulder dislocation requiring surgery and 1 periprosthetic fracture), *Mild*: 24 cases of Nerot-Sirveaux class 1) **Immediate rehabilitation: Frequency**: Rare/3 AEs, **Severity**: *Severe: 1* (pulmonary embolism), *Moderate*: 2 (glenosphere dislocation, and acromial stress fracture), *Mild*: 13 cases of Nerot-Sirveaux class 1, 2 cases of Nerot-Sirveaux class 2). **Khalil et al., 2023**^56^**:** No adverse events in any group.	**Long term:** 0 (2)	⊕⊕◯◯ Low^a,b^
**Glenohumeral Joint Immobilisation (Neutral versus internal rotation) sling position after total shoulder arthroplasty (*number of participants**** *=* ****36) ^¥^***		
***Pain (1)*** CRITICAL **Baumgarten et al., 2018**^57^ (VAS 0–100 mm (mean change)), *higher *=* worse* **Short-term:** NI, **Medium-term:** MD* *=* *−5, **Long-term:** NI	**Short term:** 0 (1), **Medium term:** 0 (1), **Long term:** 0 (1)	⊕◯◯◯ Very low^a,c^
* **Function (1)** * CRITICAL **Baumgarten et al., 2018**^57^ (DASH, 0–100 (mean change), *higher *=* worse* **Short-term:** NI, **Medium-term:** MD* *=* *6, **Long-term:** NI	**Short term:** 0 (1), **Medium term:** 0 (1), **Long term:** 0 (1)	⊕◯◯◯ Very low^a,c^
***Adverse events (1)*** IMPORTANT **Baumgarten et al., 2018**^57^ Authors reported to have assessed radiographic complications, but none were reported in the manuscript		⊕◯◯◯ Very low^a,c^
**Home versus Formal Physical Therapy (*number of participants**** *=* ****89) ^¥^***		
***Pain (1)*** CRITICAL **Chalmers et al., 2023**^55^ (VAS, 0–10), *higher *=* worse* **Short-term:** −0.20 [−1.1, 0.7], *p *=* *0.64, **Long-term:** 0.60 [−0.1, 1.3], *p *=* *0.08	**Short term:** 0 (1) **Long term:** 0 (1)	⊕◯◯◯ Very low^a,c^
* **Function (1)** * * CRITICAL **Chalmers et al., 2023**^55^ (ASES composite, 0–100) **Short-term:** −1.0 [−8.3, 6.3], *p *=* *0.80, **Long-term:** −2.0 [−21.8, 7.4], *p *=* *0.67	**Short term:** 0 (1) **Long term:** 0 (1)	⊕◯◯◯ Very low^a,c^
* **Adverse events (1)** * IMPORTANT **Chalmers et al., 2023**^55^ **Active supervised multimodal physiotherapy: Frequency**: Rare/ 4 AEs: **Severity**: *Severe*: 2 (1 axillary nerve palsy and 1 postoperative carpal tunnel syndrome), *Moderate: 2* (1 instability, and 1 stiffness) **Structured home exercise program: Frequency**: Rare/ 6 AEs, **Severity:** *Severe: 2* (postoperative ulnar neuritis), *Moderate: 2* (instability, and infection), ***Unrelated to the intervention: 2*** (intraoperative humeral fracture, 1 postoperative fall that resulted in a brachial plexitis)	**Short term:** 0 (1) **Long term:** 0 (1)	⊕◯◯◯ Very low^a,c^

**¥** **=** First intervention mentioned in the RCT is the experimental and the second is the comparator, MD = mean differences, CI = confidence interval, VAS* *=* V*isual Analogue Scale, ASES* *=* *American Shoulder and Elbow Surgeons Score, 0 (1)* *=* *no direction of effect from 1 RCT, 0 (2)* *=* *no direction of effect from 2 RCTs.

* **For** ASES, a positive MD favours the experimental group (first intervention written in the table) and negative favours control, while for both VAS pain and DASH higher* *=* *worse, therefore negative MD favoured experimental group and positive favours control, AEs* *=* a*dverse events with rare = 0–15%, (Hubal & Day, 2006), mild = bothersome but requiring no change in therapy, moderate = requiring change in therapy, additional treatment, or hospitalisation, severe = disabling or life-threatening (Calis & Young, 2004).

† All evidence was downgraded for risk of bias and imprecision. a = The RCT (s) have a moderate risk of bias, b = The total number of patients included in all the trials is less than the 400 considered as thresholds. c = The RCT (s) have a high risk of bias. CRITICAL (IMPORTANT): Considered as critical outcome based on OMERACT-OARSI recommendation.

Khalil et al.^56^ reported no adverse events. While Hagen et al.^58^ reported adverse events, the rate and pattern were however similar in both groups, as shown in Table 3.

#### Glenohumeral joint immobilisation sling position after total shoulder arthroplasty

One small RCT involving 36 participants compared the effect of immobilisation position after TSA for GHOA.^57^ The RCT compared placing the GH joint in a neutral or an internally rotated position using the Slingshot 3 sling (Arthrex, Naples, FL, USA) and the Joslin sling (Brownmed, Boston, MA, USA), respectively. The authors confirmed that the sling was applied immediately after surgery by a physician assistant and the physiotherapists were involved in monitoring and educating the patients about the appropriate sling position during the postoperative rehabilitation. Although the results favoured the neutral sling position in terms of functional improvement in the medium term (100% (binomial exact two-sided 95% CI: 25%–100%), *p* = 1.0), with very low certainty, evidence, a clear direction of effect could not be ascertained at all time-points (Table 3). This RCT was judged to have a high risk of bias (Figure 2). The available effect estimates are presented in Table 3.

The authors reported to have assessed radiographic complications, but none were reported in the manuscript; we therefore cannot ascertain the safety of the interventions.

#### Home exercise program versus physiotherapist-led care

One RCT,^55^ involving 89 participants with a mix of primary GHOA and RCA diagnoses, compared the effects of an active supervised physiotherapist-led intervention to a structured HEP after RTSA. In the physiotherapist-led group, participants received supervised physiotherapist interventions that commenced 2 weeks postoperatively, beginning with passive ROM, before progressing to active-assisted ROM and then active ROM twice a week, followed by once-weekly strengthening exercises at 3 months. The intervention was concluded when the patient demonstrated independence with a graduated strengthening program. In the HEP group, the treating orthopaedic surgeon gave the patient a rope pulley and handout illustrating the exercises to perform, which were aimed at recovering ROM and muscle strengthening. The very low certainty evidence from this RCT showed no clear evidence of direction of effect at all time-points (100% (binomial exact two-sided 95% CI: 25%–100%), *p* = 1.0) (Table 3). The RCT was judged to have a high risk of bias (Figure 2). The available effect estimates are presented in Table 3.

The frequency of adverse events was rated as ‘very rare’ in each group, with 4 events in the physiotherapist-led care group and 6 events in the HEP group (Table 3). The patterns of adverse events were similar in both groups consisting of severe events (such as nerve involvement) and moderate events (such as instability), but it was unclear whether they are linked to the surgery or the physiotherapist-led intervention or HEP.

## Discussion

### Summary of findings

This systematic review investigated the effectiveness of physiotherapist-led interventions in reducing pain and improving function in patients with GHOA including RCA. All the included RCTs focused on postoperative management, with none including patients that did not first undergo surgery.^55–58^ Only one RCT compared a physiotherapist-led intervention to a non-physiotherapist-led intervention,^55^ limiting firm conclusions regarding between-group differences especially given the evidence from this is of very low quality. Two RCTs were judged to be at high risk of bias with the other two having some concern about risk of bias. We found low-certainty evidence of no differences in effectiveness and safety of early versus delayed physiotherapist-led postoperative rehabilitation intervention for adults with GHOA, including RCA. Due to very low certainty evidence from other RCTs, no firm conclusion can be drawn regarding the effectiveness of physiotherapist-led interventions. Two ongoing RCTs investigating the effects of physiotherapist-led interventions in adults with GHOA and RCA not undergoing surgery^62,63^ may provide stronger evidence moving forward.

### Discussion of absence of evidence in non-surgical care settings

We consider here multiple issues that may contribute to the absence of RCTs investigating the effectiveness of physiotherapist-led interventions for adults with GHOA. Despite many calls for more evidence to underpin treatments for GHOA,^3,4,26^ it is possible that funders of RCTs and researchers prioritise knee and hip OA RCTs where the numbers of participants, disease burden and associated healthcare costs are the largest.^1^ Another potential reason is the lack of a strong community of GHOA researchers committed to designing and executing RCTs in GHOA. The lack of RCTs in GHOA could also be linked to current healthcare provision for GHOA and patients’ and clinicians’ attitudes and beliefs regarding physiotherapist-led interventions. For example, a recent qualitative study from the UK showed that patients with GHOA have mixed opinions and experiences of physiotherapist-led treatments,^5^ which could affect their willingness to seek, engage in or be randomised to future physiotherapist-led interventions RCTs. This could be exemplified by an RCT registered in the clinical trial registry to explore the effectiveness of manual therapy and exercise in GHOA, which was withdrawn because participants could not be recruited for 4 years, as potential participants preferred surgery.^64^ Studies in the USA have also identified many surgeons are of the opinion physiotherapist-led interventions may not be effective for GHOA, especially in moderate to severe cases.^27,28^ These clinicians’ beliefs may affect the recommendations they provide to patients, which can influence patients’ beliefs^65^ and in turn affect their willingness to be randomised in RCTs of physiotherapist-led interventions.

### Discussion of findings in postoperative care setting stage

The evidence in the postoperative rehabilitation setting is of low to very low certainty due to several methodological limitations. Firstly, the small sample size across included RCTs (only one included more than 100 participants) is not adequate to estimate reliable effect sizes, especially given the RCTs mostly comparing physiotherapist-led interventions.^66^ Secondly, in most of the RCTs, interventions in both groups are physiotherapist-led, limiting the conclusion to be drawn regarding their absolute effectiveness in the absence of a control group that did not receive a physiotherapist-led intervention.^67,68^ Furthermore, although AEs were rare in most of the RCTs, none of the RCTs attempted to examine the chronology of the AEs and attribute these to the physiotherapist-led interventions or surgery, further limiting interpretation of the safety of these interventions. Only one RCT was judged to have a low risk of selective reporting of results, with only two publishing their protocols, making it impossible to fully assess bias due to selective reporting. Therefore, the evidence does not support a confident conclusion, even regarding the relative effectiveness of these interventions.

### Comparison of findings with previous reviews

This updated systematic review builds on previous systematic reviews, such as Michener et al.^35^ which forms part of a Clinical Practice Guideline (CPG) from the American Physical Therapy Association and Karimi et al.,^34^ which, found little to no evidence on benefits of non-surgical physiotherapist-led intervention for patients with GHOA. However, these reviews included both RCTs and non-RCTs (the latter is at higher risk of bias for assessing effectiveness) and their searches are now outdated. Furthermore, Michener et al.^35^ included a non-physiotherapist-led RCT and Karimi et al.^34^ focussed on all conservative management with 18 of 19 included studies focusing on injectable therapies delivered by other healthcare professionals. Our results also reinforce the findings of systematic reviews focusing on postoperative rehabilitation after total or anatomical shoulder arthroplasty. Moffatt et al.^36^ also found very low-quality evidence showing no differences between early versus delayed rehabilitation following total shoulder replacement. In contrast, their systematic review included two RCTs which are excluded here because physiotherapists were not involved in the delivery of the intervention. However, our results contrast with the findings of Edwards et al.,^37^ which found moderate evidence from one RCT supporting that the early initiation of physical therapy (albeit delivered by exercise physiologists) promoted a significantly faster return of function and improvement in pain in the short term. The contrasting findings may relate to differing definitions for short-term outcomes (8 weeks vs. 12 weeks in the present review).

### Clinical implication of our findings

In the absence of high-quality evidence for physiotherapist-led interventions for GHOA, we therefore recommend clinical practice follow current clinical guidelines: USA GHOA-specific^32,33^ and general, such as NICE.^19^

### Future research directions

Given the searches in most previous reviews were conducted more than 5 years ago and little new evidence has since emerged, the evidence base for physiotherapist-led interventions for GHOA can be considered to be evolving extremely slowly.

Additional high-quality evidence is needed to determine the benefits and risks of physiotherapist-led interventions for people with GHOA, in all settings but especially in the non-surgical care setting. RCTs comparing physiotherapist-led interventions to attention controls or usual care should be conducted. Future RCTs should also consider health economics evaluations such as the cost-effectiveness of physiotherapist-led interventions for adults with GHOA to aid healthcare provision decision making. Additionally, with the rising prevalence of shoulder arthroplasty, there is also a need for RCTs assessing the effectiveness of physiotherapist-led interventions in patients with GHOA waiting for surgery, in terms of potentially delaying or avoiding or improving postoperative outcomes. Further, exploration of clinician and patient attitudes, beliefs and behaviours regarding physiotherapist-led interventions may give insight into the current lack of RCTs of physiotherapist-led interventions for non-surgical patients and may aid the development of an optimised intervention for testing in future RCTs.

### Strength and weakness of our review

The strengths of this systematic review include prospective registration with PROSPERO, the comprehensive search strategy without language or time restrictions, and the use of double screening, data extraction and quality assessment methods to decrease the risk of individual subjectivity and human error. We also made efforts to contact authors where it was unclear if the intervention was physiotherapist-led and where we identified published conference RCT abstracts but not full text journal articles. By providing a table of excluded and ongoing RCTs we help readers further interpret the current limited evidence base.

This review has limitations, including heterogenous data that precluded any meta-analysis. By not searching the grey literature and having insufficient RCT data to conduct funnel plot(s) we have little means to assess publishing bias.

## Conclusion

There is a very limited evidence base to draw conclusions regarding the effectiveness of physiotherapist-led interventions in improving pain and function for adults with GHOA, including RCA. As no RCT has explored the effectiveness of physiotherapist-led intervention outside of postoperative settings, the potential benefits and adverse effects of physiotherapist-led interventions beyond this context remain unknown. With low certainty, this review found no differences in effectiveness of one physiotherapist-led intervention to another, such as early versus late rehabilitation in the postoperative setting. Further high-quality, adequately powered RCTs are needed to advance this field and ensure evidence-based clinical care for people with GHOA.

## Supplemental Material

sj-docx-1-sel-10.1177_17585732261450961 - Supplemental material for The effectiveness of physiotherapy-led non-surgical and perioperative interventions for glenohumeral osteoarthritis: A systematic review Supplemental material, sj-docx-1-sel-10.1177_17585732261450961 for The effectiveness of physiotherapy-led non-surgical and perioperative interventions for glenohumeral osteoarthritis: A systematic review by Daha Garba Muhammad, Nadine E Foster, Mickylle Pelaez, Karina O’Leary, Ilana N Ackerman and Jonathan G Quicke in Shoulder & Elbow

sj-docx-2-sel-10.1177_17585732261450961 - Supplemental material for The effectiveness of physiotherapy-led non-surgical and perioperative interventions for glenohumeral osteoarthritis: A systematic review Supplemental material, sj-docx-2-sel-10.1177_17585732261450961 for The effectiveness of physiotherapy-led non-surgical and perioperative interventions for glenohumeral osteoarthritis: A systematic review by Daha Garba Muhammad, Nadine E Foster, Mickylle Pelaez, Karina O’Leary, Ilana N Ackerman and Jonathan G Quicke in Shoulder & Elbow

sj-docx-3-sel-10.1177_17585732261450961 - Supplemental material for The effectiveness of physiotherapy-led non-surgical and perioperative interventions for glenohumeral osteoarthritis: A systematic review Supplemental material, sj-docx-3-sel-10.1177_17585732261450961 for The effectiveness of physiotherapy-led non-surgical and perioperative interventions for glenohumeral osteoarthritis: A systematic review by Daha Garba Muhammad, Nadine E Foster, Mickylle Pelaez, Karina O’Leary, Ilana N Ackerman and Jonathan G Quicke in Shoulder & Elbow

sj-docx-4-sel-10.1177_17585732261450961 - Supplemental material for The effectiveness of physiotherapy-led non-surgical and perioperative interventions for glenohumeral osteoarthritis: A systematic review Supplemental material, sj-docx-4-sel-10.1177_17585732261450961 for The effectiveness of physiotherapy-led non-surgical and perioperative interventions for glenohumeral osteoarthritis: A systematic review by Daha Garba Muhammad, Nadine E Foster, Mickylle Pelaez, Karina O’Leary, Ilana N Ackerman and Jonathan G Quicke in Shoulder & Elbow
