# Supplementary material for: The effectiveness of physiotherapy-led non-surgical and perioperative interventions for glenohumeral osteoarthritis: A systematic review
Source: Shoulder Elbow. 2026 May 22:17585732261450961. Online ahead of print. doi: 10.1177/17585732261450961 (PMC13198524; doi:10.1177/17585732261450961)
Supplement: sj-docx-3-sel-10.1177_17585732261450961 - Supplemental material for The effectiveness of physiotherapy-led non-surgical and perioperative interventions for glenohumeral osteoarthritis: A systematic review [file sj-docx-3-sel-10.1177_17585732261450961.docx]

Supplementary file 3: Search filter used

| **Database** | **Search Strategy** | **Results** |
| --- | --- | --- |
| Pubmed | \| 15 \| #10 AND #11 AND #12 AND #13 AND #14 \| \| \| --- \| --- \| --- \| \| 14 \| ('activities of daily living'[MeSH Terms] OR 'shoulder pain'[MeSH Terms] OR 'function' [Title/Abstract] OR 'disability'[Title/Abstract] OR 'patient specific functional scale'[Title/Abstract] OR 'shoulder pain and disability index'[Title/Abstract] OR 'pain'[MeSH Major Topic] OR 'numeric pain rating scale'[Title/Abstract] OR 'visual analog pain scale'[MeSH Terms] OR 'disability of arm shoulder and hand'[All Fields]) \| \| \| 13 \| ('physical therapy modalities'[MeSH Terms] OR 'physiotherapy specialty'[MeSH Terms] OR 'physical therapy specialty'[MeSH Terms] OR 'physical therapy' OR 'physiotherapy' OR 'exercise therapies'[MeSH Terms] OR 'exercise therapy'[MeSH Terms] OR 'modalities, physical therapy'[MeSH Terms] OR 'physiotherapy techniques'[MeSH Terms] OR 'manual therapy'[MeSH Terms] OR 'acupuncture'[MeSH Terms] OR 'Electric Stimulation Therapy'[MeSH Terms] OR 'resistance training'[MeSH Terms] OR 'active stretching'[MeSH Terms] OR 'passive stretching'[MeSH Terms] OR 'exercise therapy' OR 'exercise therapies' OR 'modalities, physical therapy' OR 'physiotherapy techniques' OR 'manual therapy' OR 'acupuncture' OR 'electrotherapy' OR 'active stretching' OR 'resistance training' OR 'massage OR 'dry needling' OR 'thermal modalities' OR 'hydrotherapy' OR 'aquatic physiotherapy' OR 'aquatic physical therapy' OR 'stimulation, transcutaneous electric' [MeSH Terms] OR 'physical therapy modality'[MeSH Major Topic] OR 'Aquatic Therapy'[MeSH Terms] OR 'ice' OR 'heat' OR 'ultrasound' OR 'strength' OR 'athletic tape' [MeSH Terms]) \| \| \| 12 \| ('osteoarthritis'[MeSH Terms] OR 'osteoarthritis' OR 'OA' OR 'osteoarthritic' OR 'degenerative arthritis') \| \| \| 11 \| ('glenohumeral joint' OR 'shoulder joint'[MeSH Terms] OR 'shoulder'[MeSH Terms] OR 'shoulder joint'' OR 'shoulder joints' OR 'shoulder') \| \| \| 10 \| #8 NOT #9 \|  \| \| 9 \| animals [mh] NOT humans [mh] \| \| \| 8 \| #1 OR #2 OR #3 OR #4 OR #5 OR #6 OR #7 \| \| \| 7 \| groups [tiab] \|  \| \| 6 \| trial [tiab] \|  \| \| 5 \| randomly [tiab] \| \| \| 4 \| placebo [tiab] \|  \| \| 3 \| randomized [tiab] \| \| \| 2 \| controlled clinical trial [pt] \| \| \| 1 \| randomized controlled trial [pt] \| \| | 224 |
| Embase | #40. #35 AND #36 AND #37 AND #38 AND #39  #39. 'pain'/exp OR 'shoulder pain'/exp OR 'shoulder  pain and disability index'/exp OR 'disabilities  of the arm, shoulder and hand (score)'/exp OR  'disability'/exp OR 'function':ti,ab OR  'functional outcome':ti,ab OR 'visual analog  scale'/exp OR 'daily life activity'/exp OR  'shoulder pain and disability lndex' OR 'numeric  pain rating scale':ti,ab OR 'patient specific  functional scale':ti,ab OR 'disability of the arm  shoulder and hand':ti,ab  #38. 'physiotherapy'/exp OR 'physiotherapy  practice'/exp OR 'kinesiotherapy'/exp OR  'acupuncture'/exp OR 'electrotherapy'/exp OR  'resistance training'/exp OR 'exercise'/exp OR  'manipulative medicine'/exp OR 'massage'/exp OR  'hydrotherapy'/exp OR 'aquatic therapy'/exp OR  'electrostimulation'/exp OR 'transcutaneous  electrical nerve stimulation'/exp OR 'ice'/exp OR  'heat'/exp OR 'ultrasound'/exp OR 'athletic tape'  #37. 'osteoarthritis'/exp OR 'osteoarthritic' OR  'degenerative arthritis'/exp  #36. 'shoulder':ti,ab OR 'shoulder joint':ti,ab OR  'glenohumeral':ti,ab  #35. #20 NOT #34  #34. #21 OR #22 OR #23 OR #24 OR #25 OR #26 OR #27 OR  #28 OR #29 OR #30 OR #31 OR #32 OR #33  #33. 'animal experiment'/de NOT ('human experiment'/de  OR 'human'/de)  #32. (rat:ti,tt OR rats:ti,tt OR mouse:ti,tt OR  mice:ti,tt OR swine:ti,tt OR porcine:ti,tt OR  murine:ti,tt OR sheep:ti,tt OR lambs:ti,tt OR  pigs:ti,tt OR piglets:ti,tt OR rabbit:ti,tt OR  rabbits:ti,tt OR cat:ti,tt OR cats:ti,tt OR  dog:ti,tt OR dogs:ti,tt OR cattle:ti,tt OR  bovine:ti,tt OR monkey:ti,tt OR monkeys:ti,tt OR  trout:ti,tt OR marmoset*:ti,tt) AND 'animal  experiment'/de  #31. (databases NEAR/5 searched):ab  #30. 'update review':ab  #29. 'we searched':ab AND (review:ti,tt OR review:it)  #28. review:ab AND review:it NOT trial:ti,tt  #27. ('random cluster' NEAR/4 sampl*):ti,ab,tt  #26. 'random field*':ti,ab,tt  #25. nonrandom*:ti,ab,tt NOT random*:ti,ab,tt  #24. 'systematic review':ti,tt NOT (trial:ti,tt OR  study:ti,tt)  #23. 'case control*':ti,ab,tt AND random*:ti,ab,tt NOT  ('randomised controlled':ti,ab,tt OR 'randomized  controlled':ti,ab,tt)  #22. 'cross-sectional study'/de NOT ('randomized  controlled trial'/exp OR 'controlled clinical  study'/de OR 'controlled study'/de OR 'randomised  controlled':ti,ab,tt OR 'randomized  controlled':ti,ab,tt OR 'control group':ti,ab,tt  OR 'control groups':ti,ab,tt)  #21. ((random* NEXT/1 sampl* NEAR/8 ('cross section*'  OR questionnaire* OR survey OR surveys OR  database OR databases)):ti,ab,tt) NOT  ('comparative study'/de OR 'controlled study'/de  OR 'randomised controlled':ti,ab,tt OR  'randomized controlled':ti,ab,tt OR 'randomly  assigned':ti,ab,tt)  #20. #1 OR #2 OR #3 OR #4 OR #5 OR #6 OR #7 OR #8 OR  #9 OR #10 OR #11 OR #12 OR #13 OR #14 OR #15 OR  #16 OR #17 OR #18 OR #19  #19. trial:ti,tt  #18. 'human experiment'/de  #17. volunteer:ti,ab,tt OR volunteers:ti,ab,tt  #16. (controlled NEAR/8 (study OR design OR  trial)):ti,ab,tt  #15. assigned:ti,ab,tt OR allocated:ti,ab,tt  #14. ((assign* OR match OR matched OR allocation)  NEAR/6 (alternate OR group OR groups OR  intervention OR interventions OR patient OR  patients OR subject OR subjects OR participant OR  participants)):ti,ab,tt  #13. crossover:ti,ab,tt OR 'cross over':ti,ab,tt  #12. (parallel NEXT/1 group*):ti,ab,tt  #11. 'double blind procedure'/de  #10. ((double OR single OR doubly OR singly) NEXT/1  (blind OR blinded OR blindly)):ti,ab,tt  #9. (open NEXT/1 label):ti,ab,tt  #8. (evaluated:ab OR evaluate:ab OR evaluating:ab OR  assessed:ab OR assess:ab) AND (compare:ab OR  compared:ab OR comparing:ab OR comparison:ab)  #7. compare:ti,tt OR compared:ti,tt OR  comparison:ti,tt  #6. placebo:ti,ab,tt  #5. 'intermethod comparison'/de  #4. 'randomization'/de  #3. random*:ti,ab,tt  #2. 'controlled clinical trial'/de  #1. 'randomized controlled trial'/exp | 273 |
| Cochrane | #1 (shoulder joint):ti,ab,kw  #2 (glenohumeral joint):ti,ab,kw  #3 MeSH descriptor: [Shoulder Joint] explode all trees  #5 (physical therapist or physical therapy assistant):ti,ab,kw  #6 (physiotherapy or physiotherapist or physiotherapists or physical therapists):ti,ab,kw  #7 MeSH descriptor: [Physical Therapy Modalities] explode all trees  #8 #6 OR #7  #9 #5 OR #8  #10 (osteoarthritis):ti,ab,kw  #11 (degenerative arthritis or degenerative joint disease or oa):ti,ab,kw  #12 MeSH descriptor: [Osteoarthritis]  #13 #10 OR #11 OR #12  #14 #4 AND #9 AND #13  #15 (function):ti,ab,kw  #16 (pain):ti,ab,kw  #17 (disability):ti,ab,kw  #18 (shoulder pain and disability index):ti,ab,kw  #19 (disability of the arm shoulder hand):ti,ab,kw  #20 (patient specific functional scale):ti,ab,kw  #21 (visual analogue scale):ti,ab,kw  #22 (numeric pain rating scale):ti,ab,kw  #23 (activities of daily living):ti,ab,kw  #24 MeSH descriptor: [Pain Measurement]  #25 MeSH descriptor: [Pain]  #26 #15 OR #16 OR #17 OR #18 OR #19 OR #20 OR #21 OR #22 OR #23 OR #24 OR #25  #27 #4 AND #9 AND #13 AND #26 | 67 |
| CINAHL | S1  (MH "Shoulder" OR MH "Shoulder Joint+" OR MH "Glenohumeral Joint" OR Tl shoulder OR AB shoulder OR Tl shoulder joint OR AB shoulder joint OR Tl glenohumeral joint OR AB glenohumeral joint OR Tl glenohumeral OR AB glenohumeral)  S2  (MH "Osteoarthritis+" OR Tl osteoarthritis OR AB osteoarthritis OR Tl osteoarthritis or degenerative arthritis or degenerative joint disease OR AB osteoarthritis or degenerative arthritis or degenerative joint disease OR Tl osteoarthritis or oa OR AB osteoarthritis or oa)  S3  (MH "Physical Therapy+" OR Tl physical therapy or physiotherapy or rehabilitation OR AB physical therapy or physiotherapy or rehabilitation OR Tl physical therapy modalities OR AB physical therapy modalities)  S4  (MH "Activities of Daily Living+" OR MH "Pain+" OR (MH "Shoulder Pain" OR Tl pain OR AB pain OR Tl disability OR AB disability OR Tl activities of daily living or adl OR AB activities of daily living or adl OR Tl patient specific functional scale OR AB patient specific functional scale OR Tl disability of arm, shoulder and hand OR AB disability of arm, shoulder and hand OR Tl shoulder pain and disability index OR AB shoulder pain and disability index)  S5  PT (randomised controlled trial or randomized controlled trial or rct)  S6  S1 AND S2 AND S3 AND S4 AND S5 | 11 |
| Pedro | Abstract & Title: Osteoarthritis  Body Part: upper arm, shoulder or shoulder girdle  Subdiscipline: musculoskeletal  Method: clinical trial  When Searching:   - Match all search terms (AND) | 7 |
