# Supplementary material for: The effectiveness of physiotherapy-led non-surgical and perioperative interventions for glenohumeral osteoarthritis: A systematic review
Source: Shoulder Elbow. 2026 May 22:17585732261450961. Online ahead of print. doi: 10.1177/17585732261450961 (PMC13198524; doi:10.1177/17585732261450961)
Supplement: sj-docx-4-sel-10.1177_17585732261450961 - Supplemental material for The effectiveness of physiotherapy-led non-surgical and perioperative interventions for glenohumeral osteoarthritis: A systematic review [file sj-docx-4-sel-10.1177_17585732261450961.docx]

Supplementary file 4: Table of excluded and ongoing studies

| **Study first author & year** | **Title** | **Comment** |
| --- | --- | --- |
| Excluded studies | | |
| Kocic 2014 | Physical therapy in treatment of patients with glenohumeral osteoarthritis | Presented as an abstract at the World Congress on Osteoporosis, Osteoarthritis and Musculoskeletal Diseases (WCO-IOF-ESCEO 2014) |
| Di Giacomo & de Gasperis, 2015 | The role of hyaluronic acid in patients affected by glenohumeral osteoarthritis | The two arms received identical physiotherapy intervention. |
| Denard et al., 2016 | Immediate versus delayed passive range of motion following total shoulder arthroplasty. | Intervention not delivered by physiotherapy. |
| Di Giacomo & de Gasperis, 2017 | Hyaluronic Acid Intra-Articular Injections in Patients Affected by Moderate to Severe Glenohumeral Osteoarthritis: A Prospective Randomized Study. | The two arms received identical physiotherapy intervention. |
| GayretliAtan 2019 | An investigation of efficacy of manual therapy and exercise in patients with glenohumeral arthritis | RCT but it was an M.Sc. thesis; presented at conference but journal article not retrieved. |
| Di Giacomo & de Gasperis, 2021 | Glenohumeral osteoarthritis treatment with a single hyaluronic acid administration: Clinical outcomes | The two arms received identical physiotherapy intervention. |
| Edwards et al., 2021 | A randomized single-blinded trial of early rehabilitation versus immobilization after reverse total shoulder arthroplasty | Intervention delivered by exercise physiologists. |
| Larsen et al., 2022 | Are progressive shoulder exercises feasible in patients with glenohumeral osteoarthritis or rotator cuff tear arthropathy? | Only one arm. |
| **Ongoing studies** | | |
| Josefine B. Larsen | Prosthesis Versus Active Exercise Program in Patients With Glenohumeral Osteoarthritis | Status: registered with number NCT04845074 and actively recruiting with expected end date 11-2035. |
| Josefine B. Larsen | Prosthesis Versus Active Exercise Program in Patients With Rotator cuff arthropathy | Status: registered with number NCT04864158 and actively recruiting with expected end date 2038-11. |
| Edward Mulligan | Effectiveness of Manual Therapy and Exercise in Shoulder OA | Status: registered with number NCT02587559 but withdrawn because could not recruit subjects as they routinely chose surgical option. |
